# A COORDINATED ANALYSIS OF THE ASSOCIATIONS BETWEEN LONELINESS, SOCIAL ISOLATION, AND THE COGNITIVE HEALTHSPAN

**DOI:** 10.1093/geroni/igad104.0261

**Published:** 2023-12-21

**Authors:** Kathryn Jackson, Tomiko Yoneda, Jing Luo, Daniel Mroczek, Anthony Ong, Andrew Steptoe, Eileen Graham

**Affiliations:** Northwestern University, Chicago, Illinois, United States; University of California, Davis, Davis, California, United States; Northwestern University, Chicago, Illinois, United States; Northwestern University, Chicago, Illinois, United States; Cornell University, Ithaca, New York, United States; University College London, London, England, United Kingdom; Northwestern University, Chicago, Illinois, United States

## Abstract

Insufficient social connection in older adulthood is a growing public health concern. Current research suggests that loneliness and social isolation are related to consequential health outcomes in later life. There is a large and growing literature suggesting that loneliness and social isolation are associated with increased risk of dementia and mortality, but these outcomes typically have been considered separately and without controlling for competing risk factors. Using multi-state survival models which permit simultaneous estimation of transitions between several states within one model, the current study examined the extent to which loneliness, social isolation, and social asymmetry (defined as the discordance between loneliness and isolation) are associated with transitions between cognitive status categories (non-impaired, mildly impaired, severely impaired) and death. We estimated near-identical models individually across 11 independent long-term longitudinal studies (i.e., ELSA, HILDA, HRS, LISS, OCTO-TWIN, SATSA, SHARE, SHP, SOEP, MAP, MARS) using the coordinated data analysis methodological framework, representing a total sample size of over 140,000, and meta-analysis to provide precise estimates of the effects of loneliness, isolation, and social asymmetry on cognitive healthspan transitions. Preliminary findings suggest that loneliness is associated with increased risk of transitioning from non-impaired to mildly impaired cognitive functioning. Higher loneliness is associated with shorter survival time and fewer years in a healthy cognitive state. Lastly, we found that lower social asymmetry (i.e., social resilience) is associated with longer cognitive health span (more years in NCI). These results provide promising evidence suggesting that poor social relationships are robustly associated with a diminished cognitive healthspan.

